# Midline and Parasagittal Seizures are Rare in Adult Patients

**DOI:** 10.1007/s12028-019-00804-6

**Published:** 2019-08-14

**Authors:** Kapil Gururangan, Josef Parvizi

**Affiliations:** 1 Department of Neurology, Icahn School of Medicine at Mount Sinai, 1 Gustave L. Levy Place, New York, NY 10029-5674 USA; 2 Department of Neurology and Neurological Sciences, Stanford University Medical Center, 300 Pasteur Drive, Stanford, CA 94305 USA

**Keywords:** Parasagittal, Midline, Seizure, Stat electroencephalography

## Abstract

**Background:**

For decades, half of the electrodes used in traditional electroencephalography (EEG) have been dedicated to midline and parasagittal coverage. Recently, newer EEG devices have used fewer electrodes without direct coverage over the midline or parasagittal regions. However, no systematic study to date has explored the prevalence of midline parasagittal seizures, and as such the risk of missing such seizures with only ten electrodes remains unknown.

**Methods:**

We reviewed retrospective EEG data from a cohort of 300 patients at Stanford University Medical Center and determined the frequency of seizures localized to the midline parasagittal regions. We then compiled previously reported EEG cohorts that reported the prevalence of midline parasagittal seizures to validate our findings.

**Results:**

In our cohort, only two EEGs (0.66%) were identified with a midline or parasagittal seizure focus. In a subsequent study, we compiled literature evidence from 169510 EEGs and found that the prevalence of midline or parasagittal epileptic spikes/seizures was similarly less than 1%.

**Conclusions:**

Our study serves as the first to systematically explore the scope of EEG abnormalities captured exclusively by midline or parasagittal electrodes and document their very low prevalence.

## Introduction

Conventional practice of electroencephalography (EEG) in the last 60 years has relied on the standardized International 10–20 system, in which over half of the electrodes are placed over the midline and parasagittal regions of the brain [1, 2]. This convention has been followed strictly regardless of the urgency of the clinical situation. Just as the 10–20 system has been utilized in the elective and non-urgent evaluation of patients for the detection, localization, and classification of epileptiform activity to establish the diagnosis of epilepsy, the exact same system has been applied in the emergent assessment of patients with neuro-emergencies, namely status epilepticus, to guide urgent clinical decisions. While the coverage afforded by the 10–20 EEG system is valuable in detecting focal epileptic abnormalities, especially in patients undergoing monitoring for classification of their epilepsy, the added value of traditional EEG coverage in the emergent evaluation of adult patients has not been thoroughly addressed.

Multiple devices have been studied to adapt the traditional EEG infrastructure to more effectively meet the clinical needs of these emergent situations [3–5]. Such devices have frequently utilized fewer EEG electrodes to simplify and speed their application to the patient’s scalp. For instance, a new EEG system (Ceribell Rapid Response EEG) has been developed with only ten electrodes covering the lateral surface of the scalp, and ignoring the midline and parasagittal regions [6]. However, without a systematic study of the prevalence of midline parasagittal seizures, the magnitude of the risk of missing such seizures using an abbreviated EEG with fewer electrodes has remained unknown.

To fill this gap of knowledge, the present study was designed to understand (1) the frequency of focal midline or parasagittal seizures in adult EEG studies and (2) whether seizures originating from midline or parasagittal regions remain truly isolated to midline or parasagittal EEG channels.

## Methods

We reviewed the procedure notes of 300 EEGs performed in patients older than 18 years who were being evaluated in intensive care unit (ICU, *n* = 100), emergency department (ED, *n* = 100), and other inpatient wards (*n* = 100) at Stanford University Hospital [7]. EEG procedure notes for inpatient and ICU EEGs included data for the first 24 h of continuous video EEG recordings, while the EEGs in the ED were spot recordings (40–60 min). We sequentially sampled EEG recordings for which a detailed procedure note was available (e.g., including patient’s clinical history, EEG trending, video information, EEG diagnosis, and location of seizure activity) as described by Gururangan et al. [7], and did not include patients admitted to the epilepsy monitoring unit for epileptic source localization or pre-surgical planning.

**Fig. 1:**
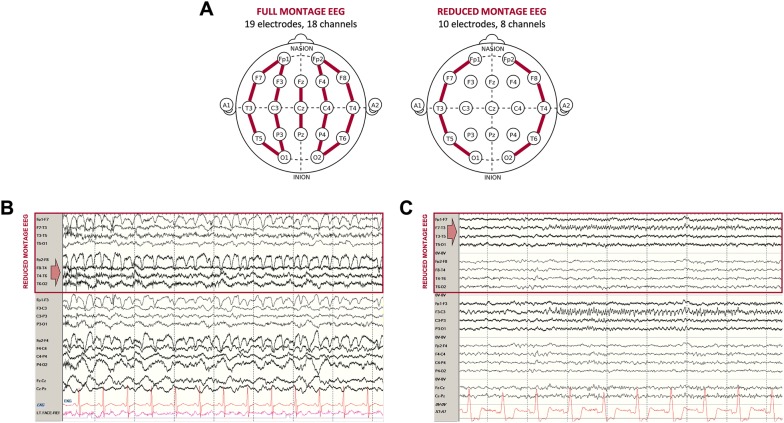
Illustration of **a** full and reduced electrode bipolar montages and samples of parasagittal seizures, indicated by red arrows, visible in right (**b**) and left (**c**) parasagittal and temporal channels (reduced electrode montage channels delineated by red boxes) (Color figure online)

We identified patients whose EEGs revealed seizure activity. To further avoid bias, we determined the presence and source of seizures (i.e., midline/parasagittal or not) by relying entirely on the details of the procedure note prepared by the clinical teams that reviewed each of the original EEGs when the EEGs were acquired. EEGs were acquired several years prior to the initiation of this study, and the original EEG readers were not aware of any discussions pertaining to our studies of reduced and full montage EEG. Based on these details, we identified seizures that were reported to be localized to midline or parasagittal regions. Each of these cases was then displayed on full and reduced electrode montage comprised of the temporal chains of the longitudinal anterior–posterior bipolar montage (Fig. 1a) to determine whether the seizure activity could be seen in the lateral channels.

To corroborate the findings from our cohort with the extant literature, we performed a brief literature review to identify past studies that reported the prevalence of midline or parasagittal epileptic abnormalities (seizures or spikes) in cohorts of patients undergoing routine monitoring with conventional EEG, again excluding patients undergoing high-density EEG monitoring to localize epileptic foci for surgical resection. For each study, we extracted the population of interest (adult vs. pediatric/neonatal), activity of interest (seizures vs. spikes), total number of EEG recordings, number of EEGs that revealed any seizures or spikes, and the number of EEGs that revealed midline or parasagittal epileptiform abnormalities. We also extracted the percentage of patients whose seizure semiology was described as having generalized onset or focal to bilateral spread, or the percentage of patients whose EEGs detected generalized seizures that might be detected in all EEG channels. From this data, we calculated the prevalence of midline or parasagittal abnormalities by pooling the cohorts reported in each study.

## Results

Among our cohort of 300 patients, 17 were found to have seizure activity on EEG. Only one of these seizures was noted in a spot EEG performed in the ED, while the rest were noted in long-term EEGs from the ICU and inpatient wards. Of these, only two were documented to have focal midline or parasagittal seizures, both in patients admitted to non-ICU wards. Both of these focal seizures were also visible in temporal EEG channels (Fig. 1) and were associated with clinical symptoms. In the first patient (Fig. 1b), ongoing focal right parasagittal seizures were associated with left face and hand twitching and rapid blinking that responded to intravenous benzodiazepines. In the second patient (Fig. 1c), evolving rhythmic activity was seen over the left parasagittal and midline regions, which spread to the left parietal and occipital regions; on the video, these seizures were associated with speech arrest and right arm and mouth movements.

We were mindful that other important EEG abnormalities such as rhythmic or periodic EEG patterns or epileptic spikes may also be of high diagnostic value in patients, and these may be missed without midline and parasagittal electrodes. To address this issue, and to validate the midline and parasagittal seizure prevalence estimate from our cohort, we performed a literature search and compiled the results of 16 pertinent studies. These prior studies were concerned with not only seizures, but also midline or parasagittal epileptiform spikes, and only one study focused solely on adult patients. (Eight included both adults and children, while seven included only pediatric patients.) The oldest published study by Pedley et al. [8], which was also conducted at Department of Neurology at Stanford University Medical Center, described a cohort of 8708 EEGs from adult and pediatric patients and reported focal epileptiform abnormalities in only 3%. Among these, midline parasagittal abnormalities were rare, occurring in only 14 (0.2%) patients (of whom only three were adults). Combining the data from all 16 studies (Table 1), the prevalence of midline or parasagittal abnormalities was 0.71% (1211 of 169510), consistent with the prevalence identified in our cohort. The percentage of seizures or spikes that could also be detected in lateral channels was not described in the prior reports; however, seizure semiologies indicative of generalized cerebral involvement or associated generalized electrographic seizures were noted in 223 of 1036 patients (22%).

**Table 1:** Summary of prior studies of midline or parasagittal seizures and spikes in adult or pediatric populations

	Population of interest	Abnormality of interest	Number of EEGs	All SZ/ES, *N* (% of EEGs)	Parasagittal SZ/ES, *N* (% of EEGs)	Associated generalized SZ*, *N* (% of parasagittal SZ/ES)
Pedley et al. [8]	A + P	ES	8708	272 (3.1%)	14 (0.2%)	5 (35.7%)
Ehle et al. [12]	P	ES	11,000	NR	37 (0.3%)	16 (43.2%)
Nelson et al. [13]	A + P	ES	8055	NR	44 (0.5%)	24 (54.5%)†
Pourmand et al. [14]	A + P	ES	7125	1166 (16.3%)	34 (0.5%)	21 (61.8%)
Molaie [15]	A	ES	1000	NR	9 (0.9%)	3 (33.3%)
Fischer and Clancy [16]	P	ES	7051	NR	21 (0.3%)	3 (14.3%)
Marshall [17]	A + P	ES	10,173	NR	43 (0.4%)	23 (53.5%)
Scher [18]	P	ES + SZ	1008	ES: NR; SZ: 92 (9.1%)	ES: 154 (15.3%); SZ: 22 (2.2%)	NR
Scher and Beggarly [19]	P	ES	1114	57 (5.1%)	21 (1.9%)	4 (19.0%)
de Paola et al. [20]	A + P	ES	14,463	1957 (13.5%)	57 (0.4%)	9 (15.8%)
Bagdorf and Lee [21]	A + P	ES	28,500	211 (0.7%)	57 (0.2%)	23 (40.4%)
Kutluay et al. [22]	A + P	ES	20,000‡	NR	35 (0.2%)	16 (45.7%)
Sanders et al. [23]	P	ES	424	228 (53.8%)	21 (5.0%)	NR
Yong et al. [24]	A + P	ES	7929	NR	17 (0.2%)	12 (70.6%)
Vendrame et al. [25]	P	ES	12,000	NR	69 (0.6%)	15 (21.7%)
Datta et al. [26]	P	ES	30,760	NR	576 (1.9%)	49 (39.8%)§
This study [7]	A	SZ	300	17 (5.7%)	2 (0.66%)	0 (0%)
Summary			169,510		1211 (0.71%)	223 (21.5%)

*A* adult, *EEG* electroencephalogram, *ES* epileptiform spikes, *NR* not reported, *P* pediatric, *SZ* seizures

*Includes both clinical generalized seizures (i.e., generalized motor [tonic and/or clonic, myoclonic, atonic] and non-motor [absence] and focal to bilateral tonic–clonic per ILAE 2017 classification [27]) and electrographic generalized seizures

†Of 34 patients were noted to have seizures, 11 had multiple seizure types, and the precise number of purely focal seizures was not reported; therefore, we used only the reported number of tonic–clonic seizures

‡Kutluay and colleagues reported “~ 20,000 EEGs,” so that estimate was used for calculations

§The authors used 123 patients out of 576 patients with midline spikes as the study group in their case–control study, and since the prevalence of generalized seizures was not reported among the excluded patients, the percentage was calculated with the study group sample size as the denominator

## Discussion

Although high-density EEG provides superior spatial precision for epileptic source localization and pre-surgical planning, emergent EEG evaluation should value timely acquisition of diagnostic information and adequate sensitivity for the most common abnormalities. The majority of emergent EEGs, especially in the ICU and ED, are ordered to evaluate for status epilepticus, particularly non-convulsive status epilepticus, as the cause of altered mental status. In these settings, it remains to be explored if focal and confined seizures in the midline parasagittal region can account for gross alterations of consciousness without any spread to other cortical or subcortical areas covered by the lateral chains of the 10–20 EEG system. However, there is some evidence in the literature suggesting that seizures originating from midline parasagittal regions, when generalized, can be detected from lateral chains of the EEG. Seminal research by Tükel and Jasper reported 31 patients with parasagittal lesions, of whom 26 also displayed synchronous rhythmic spike and wave patterns that were visible in temporal electrodes bilaterally [9]. There is also evidence that focal seizures confined to midline parasagittal region cause focal problems instead of alteration of the patient’s state of consciousness. For instance, in a study by Kennedy (1959) reported electrographic activity associated with medial epileptogenic lesions, and all seven reported patients displayed focal clinical symptoms indicative of seizure activity involving sensory and motor regions [10].

 Despite the remaining unknowns, our own study in combination with the literature evidence—albeit drawn from different patient populations—clearly confirms that focal midline or parasagittal seizures are rare in adults undergoing EEG monitoring. This claim does not pertain to infants or neonates who may have primarily focal midline seizures [11]. Moreover, our findings do not discount the importance of midline and parasagittal coverage in patients with structural lesions in these regions that may be causing focal seizures. Lastly, we recognize the importance of a greater number of EEG electrodes for more precise anatomical localization of the source of ictal activity or patterns of sleep architecture; therefore, our claim does not discount the importance of high-density EEG recordings in targeted populations when identification of the source of epileptic activity is important. With these boundaries in mind, we hope our findings fill an important gap of knowledge and serve as a systematic study of the rare prevalence of midline parasagittal seizures captured in the traditional EEG practice.
